# Respiratory Syncytial Virus (RSV) Infection in Elderly Mice Results in Altered Antiviral Gene Expression and Enhanced Pathology

**DOI:** 10.1371/journal.pone.0088764

**Published:** 2014-02-18

**Authors:** Terianne M. Wong, Sandhya Boyapalle, Viviana Sampayo, Huy D. Nguyen, Raminder Bedi, Siddharth G. Kamath, Martin L. Moore, Subhra Mohapatra, Shyam S. Mohapatra

**Affiliations:** 1 Department of Internal Medicine, James A. Haley Veterans Affairs Hospital, Tampa, Florida, United States of America; 2 Division of Translational Medicine and Nanomedicine Research Center, Department of Internal Medicine, Morsani College of Medicine, University of South Florida, Tampa, Florida, United States of America; 3 Department of Pediatrics, Emory University, Atlanta, Georgia, United States of America; 4 Children's Healthcare of Atlanta, Atlanta, Georgia, United States of America; 5 Department of Molecular Medicine, Morsani College of Medicine, University of South Florida, Tampa, Florida, United States of America; Johns Hopkins School of Medicine, United States of America

## Abstract

Elderly persons are more susceptible to RSV-induced pneumonia than young people, but the molecular mechanism underlying this susceptibility is not well understood. In this study, we used an aged mouse model of RSV-induced pneumonia to examine how aging alters the lung pathology, modulates antiviral gene expressions, and the production of inflammatory cytokines in response to RSV infection. Young (2–3 months) and aged (19–21 months) mice were intranasally infected with mucogenic or non-mucogenic RSV strains, lung histology was examined, and gene expression was analyzed. Upon infection with mucogenic strains of RSV, leukocyte infiltration in the airways was elevated and prolonged in aged mice compared to young mice. Minitab factorial analysis identified several antiviral genes that are influenced by age, infection, and a combination of both factors. The expression of five antiviral genes, including pro-inflammatory cytokines IL-1β and osteopontin (OPN), was altered by both age and infection, while age was associated with the expression of 15 antiviral genes. Both kinetics and magnitude of antiviral gene expression were diminished as a result of older age. In addition to delays in cytokine signaling and pattern recognition receptor induction, we found TLR7/8 signaling to be impaired in alveolar macrophages in aged mice. *In vivo*, induction of IL-1β and OPN were delayed but prolonged in aged mice upon RSV infection compared to young. In conclusion, this study demonstrates inherent differences in response to RSV infection in young vs. aged mice, accompanied by delayed antiviral gene induction and cytokine signaling.

## Introduction

Respiratory syncytial virus (RSV) infections result in an estimated 10,000 deaths per year in persons over the age of 65 [1], [2]. RSV employs multiple defenses against the innate and adaptive antiviral response [3]–[6] and respiratory infections exacerbate pulmonary stress in immunocompromised and atopic individuals by increasing airway resistance and potentiating hypoxemia [7], [8]. The virus alters cytokine production and can result in chronic inflammation, respiratory failure, and death [2], [9]. Among individuals over the age of 65, RSV has a reported mortality rate of 8% and 78% of deaths with underlying respiratory and pulmonary deaths are RSV-associated [10], [11]. Numerous reports have identified an age-related decline in both adaptive and innate immune responses to infections [12], [13], which has lead to the term ‘immunosenescence.’ In addition to declines in immune responses, the elderly have increased pro-inflammatory cytokine levels including IL-6 and TNF-α [14] but their responses to viral infections remain impaired through unclear mechanisms [15]. Although elderly and immunocompromised individuals remain at high risk for severe RSV disease, there is currently no effective vaccine or prophylactic available to these populations.

Impaired innate pattern recognition receptor (PRR) signaling was recently correlated with increased airway mucin expression in response to the non-mucogenic RSV strain A2, particularly in mice lacking Toll-like receptors (TLRs) 3 and 7 [16]. This suggests that adequate innate signaling and responses are required for preventing RSV-induced mucogenesis. Two alternative PRR pathways, involving the Nod-like receptors (NLRs) and retinoic-inducible gene (RIG)-like receptors (RLRs), lead to production of antiviral mediators such as IL-1β or TNF-α and Type I interferons, respectively. In addition to proinflammatory cytokines IL-6, TNF-α, and IL-1β, the secreted phosphoprotein known as osteopontin (OPN) is also linked to leukocyte chemotaxis and anti-pathogen activities [17]–[20]. Despite the established association of increased age with chronic proinflammatory cytokine profiles, the impact of age-associated hyperinflammation remains incompletely studied in the context of RSV-induced disease. Decline in PRR function [21] or aberrant expression of inflammatory cytokines due to age may provide insight as to why the elderly develop RSV-induced immunopathology.

Aged mouse models have been infrequently used in RSV investigations [22], [23]. In the few published studies performed with aged mice, viral titers obtained from lung homogenates were not significantly different between the young and aged at the peak of infection [22], [24] suggesting RSV-associated morbidity among the elderly could be due to enhanced immunopathology rather than increased viral loads. Similarly, aged cotton rats (>9 months) infected with Long RSV strain had peak viral lung titers and histopathology comparable to those seen in young (<2 months) infected cotton rats. They also demonstrated delayed viral clearance at later stages of infection [25]. Most cytokines, including IFN-γ, IL-4, IL-10, IL-6, and the chemokine MCP-1, had similar rates of induction upon RSV A2 infection for young and aged cotton rats. The lack of observable differences between age groups in gene expression and histopathology may be attributed to the use of laboratory RSV strains, A2 and Long, which do not induce substantial histopathology or mucogenesis in BALB/c mice [26]. Other recent investigations on aging and RSV A2 disease examine the age-associated changes in adaptive immune responses, such as reduced neutralizing antibody production in response to RSV vaccine candidates [24] or hampered CD8 T-cell responses despite functional capacity to secrete cytokines [13], [27]. However, age-related changes to the early, innate antiviral responses to early RSV remain insufficiently studied.

In these experiments, we sought to study RSV-induced immunopathology as it relates to aging innate responses and aberrant cytokine regulation by comparing the A2 laboratory strain with a mucogenic clinical isolate, 2–20 and with rA2-L19F, a recombinant strain of A2 containing the fusion protein of Line 19. Strains 2–20 and rA2-L19F induce goblet cell metaplasia and mucogenesis [16], [28] and are useful for inducing severe RSV bronchiolitis and disease pathology in the mouse model [26], [29].

We hypothesized that aging results in diminished PRR and antiviral signaling, which increases cell infiltration within the airway and delays leukocyte clearance. These occur independently of RSV burden at peak of infection, which remains largely unchanged due to age. In order to elucidate the molecular basis for increased RSV morbidity and mortality among the elderly, we infected young and aged mice with the mucogenic strain of RSV and identified the age-specific deficits in the innate antiviral system including increased cell infiltration and altered cytokine expression.

## Materials and Methods

### Mice

Old (19–21 mos) and young (2–3 mos) BALB/c mice were purchased from Charles River Laboratories (Wilmington, MA) under a contractual arrangement with the National Institute on Aging. OPN-/- or knockout (OPN KO) were purchased from Jackson Laboratory (strain B6.Cg-Spp1tm1Blh/J) and were backcrossed with wildtype (WT) C57BL/6 that were also purchased from Jackson Laboratories. All animal work was approved by and performed in accordance with the policies of the University of South Florida Institutional Animal Use and Care Committee.

### Cell culture

HEp-2 cells (CCL 23; American Type Culture Collection, Rockville, MD) were used to propagate RSV and for viral plaque assays. This cell line was serially passaged in DMEM supplemented with 5% fetal bovine serum (FBS). Cells were routinely tested for mycoplasma using the LookOut Mycoplasma PCR Detection Kit (Sigma). Opti-MEM supplemented with 2% FBS was used to maintain HEp-2 cell cultures during virus propagation and semi-clarification.

### Respiratory syncytial virus propagation and plaque assay

RSV A2, an A subtype RSV, was obtained from the ATCC (Cat. No. VR1302). Working stocks of this virus were prepared by infecting semiconfluent monolayers of mycoplasma-free HEp-2 cells. When the infected monolayers exhibited approximately 80% syncytia formation and substantial cytopathic effects, the cells and medium from the monolayers were collected, pooled, and clarified by centrifugation (20 mins at 1000×g at 4°C). The resulting supernatant was aliquotted, snap frozen on dry ice, and stored at −80°C until use. UV-inactivation of RSV was performed by irradiating aliquots of RSV A2 with 1200 mJ of UV for 20 mins using a Stratalinker. Mock-infection media was obtained by growing HEp-2 at semiconfluency for 2 ½ days with no infection and cell culture supernatant was clarified, aliquotted, and snap frozen in a similar method to the RSV stocks. The rA2-L19F and clinical isolate 2–20 were propagated as described [29]. The virus strains were propagated in HEp-2 in an identical manner as A2 and used to assess age-specific mucogenesis and cell infiltration in BALB/c mice.

### Histopathological analysis of A2, 2-20- and rA2-L19F-infected mice

BALB/c mice were intranasally infected with 10^5^ plaque forming units (pfu)/mouse of either A2, 2-20, or rA2-L19F and euthanized 5 or 8 days post-infection (dpi). Infectious dose was chosen based on references that previously characterize mucogenic strains of RSV in the mouse model [26], [29]. To compare infection of WT C57BL/6 with OPN-/-, a higher infectious dose (1×10^6^ pfu/mouse) was used because previous reports indicate C57BL/6 are resistant to RSV infections [30]. After euthanasia, chest cavity was opened and the lungs were gently inflated intratracheally with 4°C 4% paraformaldehyde in PBS, removed and immersed in 4% paraformaldehyde at 4°C for an additional 6 hrs. An equal volume of 30% sucrose in PBS was added and tissues were fixed overnight at 4°C. The next day, the solution was replaced with 30% sucrose in PBS and tissues were kept at 4°C for an additional 24 hrs. Fixed tissues were gently blotted before OCT embedding and snap freezing on dry ice. Four 5 µm sections from a single mouse were placed on a single slide and stained with periodic acid-Schiff (PAS) reagent which stains glycoproteins and mucins (ThermoFisher). PAS-stained lungs were viewed with a DP72 digital camera on an IX71Olympus fluorescence microscope; five images were collected from four lengthwise sections per mouse. To quantify cell infiltration, lung images were converted to binary 8-bit formats and maximal points were quantified in areas surrounding bronchioles after noise and background were minimized using the Image-based Tool for Counting Nuclei (ITCN) plug-in with ImageJ. Five bronchioles were quantified per field of view and at least six images were collected from a single mouse. Averages of counts from each mouse (n = 4) were used to generate box plots and analyzed with Minitab software (Minitab Inc., State College, PA) with ANOVA 2-way analysis. PAS-staining was estimated using Color Deconvolution analysis and custom vector settings that were obtained through single-stain vector analysis (http://www.dentistry.bham.ac.uk/landinig/software/cdeconv/cdeconv.html). Individual vectors were then used to approximate the area of PAS-stained or hematoxlyin-stained tissue regions, yielding a percentage of PAS/hematoxylin. The complete experiment and histological analysis was performed in duplicate.

### Assessment of RSV infection in the lung

A portion of the whole lung homogenate from each mouse was used in ELISAs to measure cytokines while the remaining homogenate was used to obtain viral titers using the plaque immunostaining method as previously described [31]. Briefly, snap frozen lungs were homogenized on ice in 5 volumes (wt/vol) of prechilled FBS-free OptiMEM using glass Dounce homogenizers. Tissue debris was pelleted by centrifugation at 4°C for 10 min at 300×g and supernatants were immediately serially diluted in FBS-free medium. Serial dilutions were used to infect 80%-confluent HEp-2 cells in a 24-well plate and after an hour of infection at 37°C with frequent rocking, the medium was replaced with complete overlay growth medium containing 0.8% methylcellulose, penicillin/streptomycin/amphotericin B. After five days of growth, plaques were immunostained with monoclonal mouse anti-RSV F antibody (AbDSerotec, MCA490), followed by a horseradish peroxidase-conjugated anti-mouse secondary antibody, and visualized with 4CN substrate (Kirkegaard and Perry Laboratories). The remaining lung homogenates, plasma samples, and BALFs were analyzed by ELISA for mouse IL-6 (eBioscience, 88-7064), IL-1β (eBioscience, 88-7013), and osteopontin (OPN; Abcam, ab100734). Quantitative RT-PCR for detection of RSV N and RSV F genes confirmed RSV infection in all lung tissues examined as previously described [32].

### Gene profiler RNA expression analysis

Mice were intranasally infected with 10^6^ pfu/mouse of the non-mucogenic strain A2 and mice were euthanized at 1, 3 or 5 dpi. The moderate inoculum dose was selected based on multiple references that described intranasal infection with 10^6^ pfu of RSV A2 per mouse is sufficient to induce lung disease and obtain plaque titers from lung homogenates [24], [33]. Total RNA was isolated from lung tissue and antiviral gene expression was quantitated using RT^2^-PCR Profiler Arrays (PAMM-122; SABiosciences) in a BioRad CFX96 real-time PCR system. C_t_ values were obtained using a constant baseline threshold for all PCR runs and samples were run in triplicates using the manufacturer's thermocycler conditions. Five endogenous expression controls provided by the array (Hprt, Hsp90ab1, Gusb, Gapdh, and Actb) were used to calculate the arithmetic mean which was then set as the C_t_ value for normalization. Scatter-plots were generated using RT^2^PCR Profiler Data PCR array analysis software and data quality checks were performed. Gene expression changes >2-fold were separately analyzed using GeneGo (Thermo Scientific Inc.) software and GeneMania predictive interaction pathway maps [34]. Biologically relevant networks were assembled using genes associated with >2-fold increase in young mice as compared to aged mice on 1 dpi. Linkages are color coded and indicated co-expression (purple), colocalization (light blue), predicted interactions (orange), shared protein domains (light yellow), or other undefined relationships (gray). Individual gene expression charts were generated to demonstrate the difference in fold-change increases within age groups along a timecourse. Experimental gene expression was defined as the Delta-Delta cycle threshold (ΔΔCt) and calculations were normalized to five endogenous housekeeping genes and in mock-treated, age-matched controls. Heatmaps encompassing the complete 84-gene PCR array were generated using GENE-E software (http://www.broadinstitute.org/cancer/software/GENE-E/). Gradients from blue to red indicate minimum to maximum expression of the indicated genes, within each row. Columns indicate the aged and young groups at 1 dpi, 3 dpi, or 5 dpi. One minus the Pearson's correlation value was used for hierarchical clustering; columns indicate timepoint while rows indicate gene examined. Individual mouse RNA array analysis was performed in a single experiment with three different time points (n = 3 mice/group) and gene expression data is displayed as mean values +/- SEM; statistical significance was assessed using 2-way ANOVA.

### Minitab statistical analysis for design of experiments (DOE)

The first Delta Ct (ΔCt) was defined as the difference between the average Ct values of the five endogenous control genes (Hprt, Hsp90ab1, Gusb, Gapdh, and Actb) and the Ct value of the target gene for an individual mouse (n = 3). The ΔCt was designated as the response variable and two processing conditions, either age or RSV exposure, were defined for each sample in a two-level factorial DOE analysis with the terms: age, infection, or age*infection. Factorial fit results were derived using alpha  = 0.05, such that p-values <0.05 were considered significant. Estimated effects and coefficients were tabulated for each of the 84 antiviral genes.

### Quantitative PCR analysis

Real-time PCR performed on cDNA was used to quantitate the expression of RSV N, RIG-I, IL-1β, IL-6, osteopontin (OPN) using DyNAmo Flash SYBR Green qPCR kits. BALB/c young and aged mice were infected with either RSV A2 at dose of 10^6^ pfu/mouse (n = 4) or either rA2-L19F or 2-20 RSV with a single intranasal infection dose of 10^5^ pfu/mouse (n = 4). Inoculum doses used were based on published references that characterize moderate (10^6^ pfu/mouse) RSV A2 infections in the mouse yield quantifiable plaque titers and result in significant lung disease [24], [26], [29] while low dose of mucogenic RSV strains (10^5^ pfu/mouse) is sufficient to induce disease. RSV A2- and rA2-L19F-infected mice were euthanized at days 1, 3, 5, and 8 while tissues from 2-20-infected mice collected on days 1, 4, and 8. These time points were chosen because RSV A2- N levels peak at 3 dpi and 2-20-RSV N levels peak at 4 dpi. Experiments were performed in triplicate (n = 4 per group). Primer sequences for qPCR were obtained from published references and melting curve analysis was performed to confirm specificity. Primers used for amplification of gene transcripts were as follows: IL-1β For 5′-GAA GAT GGA AAA ACG GTT TG-3′, IL-1β Rev 5′-GGA AGA CAC GGA TTC CAT GG-3′; IL-6 For 5′-GTC TAT ACC ACT TCA CAA GTC GGA G-3; IL-6 Rev 5′-GCA CAA CTC TTT TCT CAT TTC CAC-3′; OPN For 5′- AGC AAG AAA CTC TTC CAA GCA A-3′, OPN Rev 5′- GTG AGA TTC GTC AGA TTC ATC CG, Line 19 RSV N For: 5′-CAT CTA GCA AAT ACA CCA TCC A-3′, Line 19 RSV N Rev: 5′-TTC TGC ACA TCA TAA TTA GGA GTA TCA A-3′, MUC5AC For: 5′-CCATGCAGAGTCCTCAGAACAA, MUC5AC Rev: 5′-TTACTGGAAAGGCCCAAGCA; additionally, IDT PrimeTimeTM qPCR primers were used with the following sequences: CD44 For 5′-ACA CCT CCC ACT ATG ACA CAT-3′ and CD44 Rev 5′-TCA CGG TTG ACA ATA GTT ATG GT-3′. A master mix solution was prepared as follows: 2.5 µl of 2X DyNamo Color Flash SYBR master mix, 0.15 µl of the appropriate forward and reverse primers (stock concentration, 10 µM) and 1.2 µl of H_2_O per sample. 1 µl diluted cDNA and 4 µl of the master mix solution were added to each well of a 384 well optical reaction plate (Thermo Fisher). All samples, including a H_2_O control, were run in four replicates using the BioRad CFX384™ Real-time PCR Detection system. The data were analyzed using ΔCt and ΔΔCt calculations and expression of all genes was normalized to mouse endogenous control hypoxanthine-guanine phosphoribosyltransferase: HPRT For 5′-GCT GAC CTG CTG GAT TAC ATT AA-3′, HPRT Rev 5′-TGA TCA TTA CAG TAG CTC TTC AGT CTG A-3′.

### Collection of alveolar macrophages and ex vivo macrophage stimulation

Bronchoalveolar lavage (BAL) was performed on an uninfected group of aged and young mice for isolation of alveolar macrophages. Immediately after euthanasia, blood was collected via cardiac puncture and 1 mL of room-temperature sterile phosphate buffered saline (PBS) containing 5 mM EDTA was injected into the lungs through the trachea, avoiding contamination with red blood cells, and the fluid was recovered. This was repeated twice and BAL fluids (BALFs) were pooled and kept on ice. BALFs were centrifuged at 4°C for 10 mins at 300×g and resuspended in pre-warmed (37°C) 10% RPMI media. Cells were counted with a hemacytometer and seeded at a density of 8×10^5^ cells per well in a 24-well plate. Cells were allowed to adhere for 2 hours at 37°C then washed twice with 37°C PBS. An extra well from each age group was trypsinized to count the remaining adherent alveolar macrophages. Equal numbers of adherent cells were incubated with 2.5 µg/ml of the TLR7/8 ligand, R848. After 20 hrs, supernatants were collected and analyzed for secretion of IL-1β and IL-6 to examine TLR7/8 activation. Experiments were performed in triplicate and data is represented as mean +/- SEM. Statistical significance was determined by 2-way ANOVA, p<0.05.

### Immunohistochemical staining

Lung sections were probed for IL-1β and OPN with goat anti-mouse primary antibodies (R&D AF401-NA and AF808, respectively) or goat IgG control antibody then visualized with nickel-3,3′-diaminobenzidine tetrahydrochloride (DAB) reagent (ABC Vectastain and DAB kit, Vector Labs); sections were counterstained with hematoxylin and eosin before dehydration, clearing, and mounting of coverslips. To obtain the percentage of hematoxylin-stained cells in the presence of nickel-DAB immunostaining, lung sections were analyzed with ImmunoRatio ImageJ plugin after calibrating to a blank-field correction image and adjusting threshold. Five bronchioles were quantified per field of view and at least 6 images were collected from a single mouse. Percentages from each mouse (n = 4) were used to generate box plots and analyzed with Minitab software with 2-way ANOVA. Prior to collection of images, background and nonspecific nickel-DAB staining was subtracted through comparison of nonspecific goat Ig control antibody. A polyclonal goat antibody for RSV (Millipore, Ab1128) was used to detect RSV antigens in lung sections, followed by indirect immunofluorescence using secondary anti-goat IgG Alexa Fluor-555 conjugated antibody. Sections were mounted with 4′,6-diamidino-2-phenylindole (DAPI)-containing anti-fade mounting media (SouthernBiotech, Dapi-Fluoromount G) and, after background and blackfield corrections, a minimum of 10 images at 20X magnification were collected per mouse (n = 4) with the DP72 digital camera on an IX71Olympus fluorescence microscope on both fluorescent channels. Single-channel images were used to quantify the number of DAPI-stained nuclei or RSV-positive cells using ITCN ImageJ plugin using differential values for diameters of nuclei versus individual cells, yielding a percentage of RSV-positive cells from total cells per field of view.

### Statistical analysis

For PCR array analysis, n = 3 and a constant C_t_ threshold was applied to each sample during data analysis, as per the manufacturer's recommendation, and entire experiment was performed once. In all following mice experiments that analyzed plaques, gene expression, or cytokines, n = 4-6 mice per group and mock-infected groups were harvested on day 5 and 8; cytokine data were compared to the mock-infected groups harvested on the same day as experimental group. Experiments were performed in triplicate and data presented are the mean, +/- SEM. Statistical significance was determined using ANOVA, p<0.05.

## Results

### Mucogenic RSV induces differential leukocyte infiltration in aged mice

**Figure 1:**
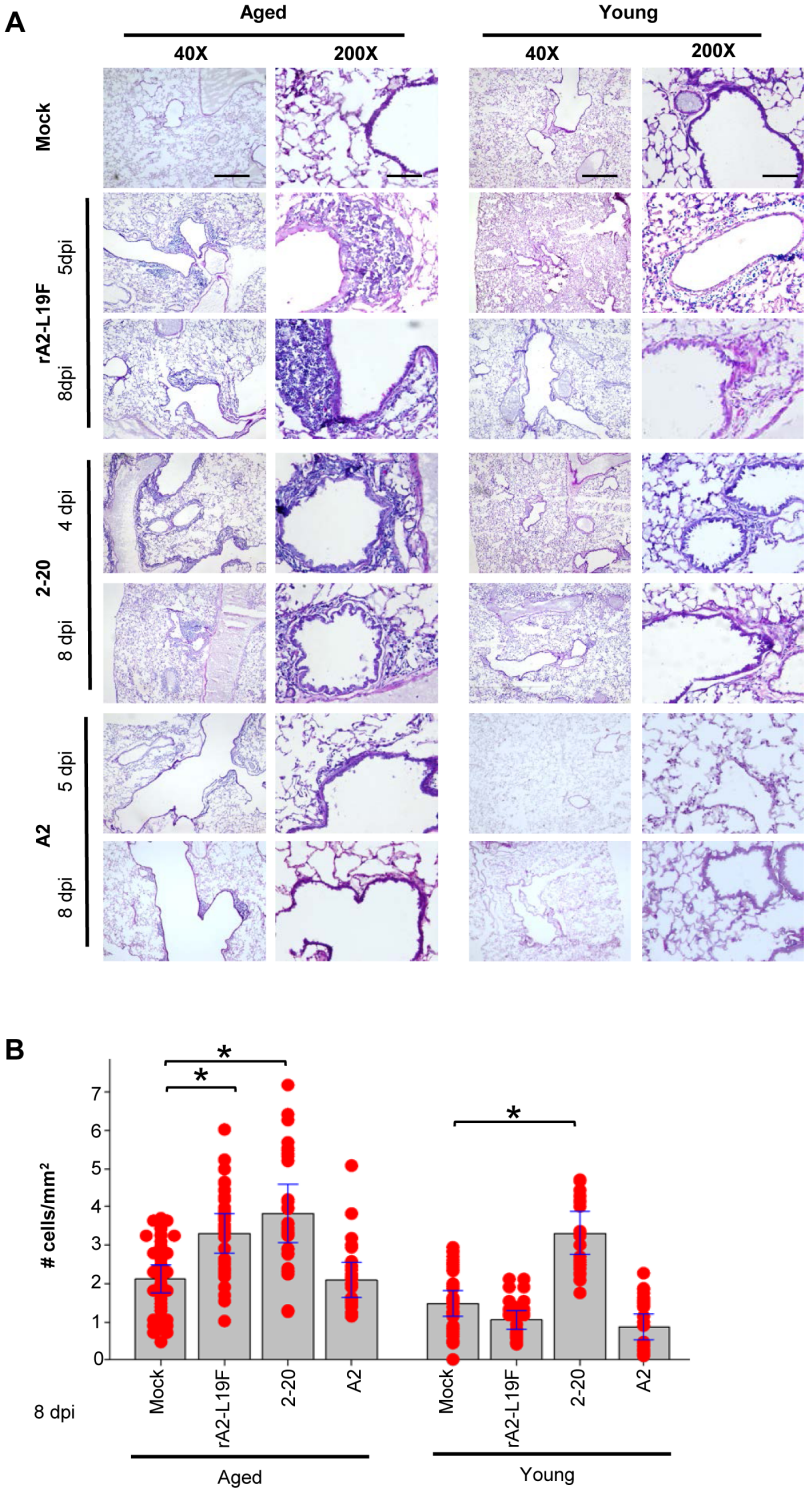
Aged mice have enhanced pathology upon infection with mucogenic RSV. (A) Young and aged mice were infected with a dose of 10^5^ pfu/mouse of either RSV 2–20 or rA2-L19F or 10^6^ pfu/mouse of A2 for comparison. Lungs were collected on days indicated and sectioned at 5 µm, air dried, then stained with PAS and counterstained with hematoxylin and eosin. Representative images of either aged or young mice are shown in two main columns, with rows indicating mock-infection or infection with either rA2-L19F, 2–20 or A2; scale bars indicate 200 µm (first column, 40X) or 40 µm (second column, 200X), respectively. At 40X magnification, hematoxylin stained nuclei were quantified with ImageJ analysis. Five bronchioles were quantified per field of view and at least 8 images were collected from a single mouse (n = 4/group). (B) Individual dots in the box plot represent a mean infiltration density count (# cells/mm^2^) from each mouse within a mock-infected or group infected with RSV. Significance was obtained through ANOVA and Fisher's exact post hoc test, p<0.05. Histological analysis was repeated in two separate experiments.

We infected young and aged BALB/c mice with the mucogenic RSV strains rA2-L19F and 2-20 and laboratory strain A2 to observe how aging affects mucogenesis and cell infiltration. Upon intranasal infection, cell infiltration was found to be greatest among aged mice between 4-5 dpi with rA2-L19F or 2-20, however pathology was less apparent in A2 infection (Fig. 1A). The pathology was still visible on day 8 in aged mice while infiltration was diminished in young mice. To compare the cellular clearance at late stages of infections, hematoxylin stained nuclei were quantified with ImageJ analysis and represented as infiltration density box plots at 8 dpi (Fig. 1B). Infection with RSV rA2-L19F and 2-20 resulted significant cell infiltration on 8dpi in aged mice, while young mice infected with 2-20, but not rA2-L19F, had significant cellular densities. Upon histological analysis of lungs obtained from rA2-L19F and 2-20 infected mice at 4 and 8 dpi, both young and aged mice have dense cell infiltration at 4 dpi (data not shown). Although not statistically significant, 2-20 infections in aged mice had a trend of delayed cellular clearance at 8 dpi as compared to young. In contrast, A2 did not induce substantial mucin production which was examined using PAS-stained lungs and color deconvolution analysis (data not shown). Although not statistically significant, we observed a trend of reduced mucin staining in the lungs of infected aged mice as compared to young and this was supported further with gene expression of MUC5AC (data not shown).

**Figure 2:**
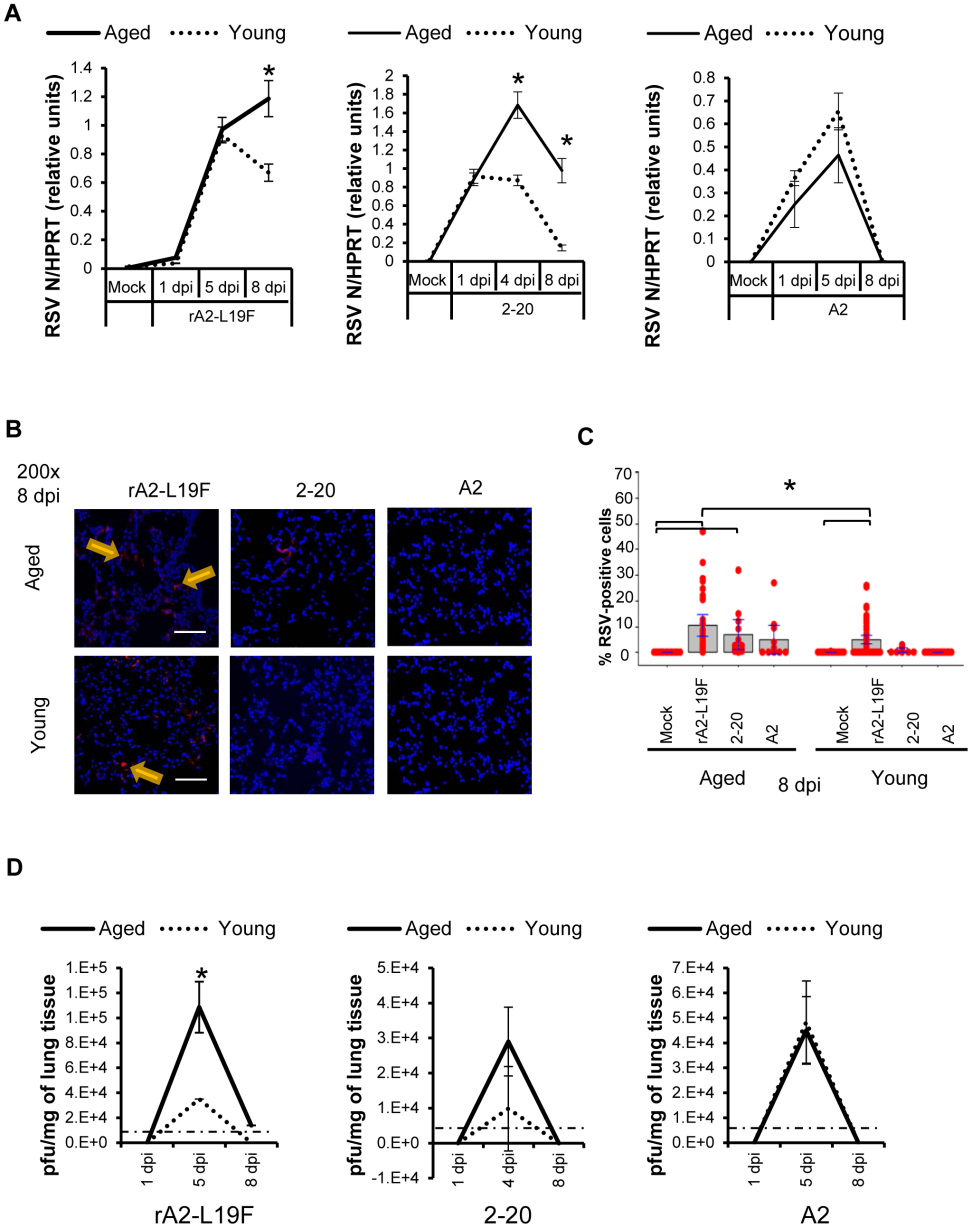
Age-differential infectivity with mucogenic RSV. Young and aged BALB/c mice (n = 4/group) were intranasally infected with a dose of 10^6^ pfu/mouse of A2 or 10^5^ pfu/mouse of rA2-L19F or 2-20 and total lung RNA was collected on time points indicated. (A) RNA transcripts of RSV N were analyzed with qRT-PCR and represented as a relative ratio of target gene expression to endogenous mouse HPRT with arbitrary units. Significance was determined with ANOVA, p<0.05. (B) Lung sections were stained for RSV antigens using polyclonal antibody and Alexa Fluor 555-conjugated secondary antibody. Representative images shown are 200X magnified merged images of DAPI-(blue) and RSV-positive (red) from 8 dpi with either rA2-L19F, 2-20, or A2 infections in both young and aged mice. (C) Immunofluorescence analysis was performed using images at 200X magnification, with a minimum of 10 images per lung section per mouse (n>4). RSV-positive cells were enumerated using ITCN ImageJ analysis. DAPI- and RSV-positive cells were counted separately to provide the percentage of RSV-positive cells, which is illustrated in the individual dot plot shown. Statistical significance was determined using one-way ANOVA and Fisher's exact post hoc test, p<0.05. (D) Portions of the right lung tissues from either rA2-L19F, 2-20-, or A2- infected mice were gently homogenized in 5X (wt/vol) of culture media before being serially diluted and used to infect semi-confluent HEp-2 cells in 24-well plates. Plaques were obtained using immunostaining technique and visualized with 4CN peroxidase substrate. Dotted horizontal lines at 5×10^3^ indicates the limit of detection using immunostaining technique. Plaques were obtained from individual mice within a group (n>4) and experiments were repeated twice.

In contrast to previous studies with RSV A2 [22], rA2-L19F and 2-20 infections in aged mice resulted in elevated expression of RSV N at 4 dpi for 2-20, and remained elevated on 8 dpi for both rA2-L19F and 2-20 (Fig. 2A). The RSV N levels measured by qRT-PCR were not statistically different between the two age groups on days 1, 5 or 8 dpi with RSV A2. The lungs were then stained for RSV antigens and examined with immunofluorescence analysis (Fig. 2B). Infection with rA2-L19F resulted in more RSV-positive cells on 8 dpi than either RSV strain 2-20 or A2, which had little to no infection visible. Images were quantified and indicated as box plots (Fig. 2C). RSV rA2-L19F and 2-20 infections were prolonged to 8 dpi; we observed A2 was cleared in both age groups by 8 dpi and is in agreement with previous studies[22]. Plaque titration was performed on lung tissues and compared between strains (Fig. 2D). Similar to that observed in RSV N expression, aged mice had more plaques than young mice; however, no difference was observed between 2–20 and A2. It is noted that rA2-L19F was previously reported to grow more efficiently in HEp-2 cells in comparison to A2[29] and 2-20[26].

### Aging leads to altered kinetics of antiviral gene expression

We initially wanted to identify gene targets that are predominantly influenced by age and not necessarily RSV pathogenicity. Gene expression profiles were generated using the RT^2^-PCR Profiler arrays, which screen 84 mouse-specific antiviral receptors, mediators, and signaling components. The baseline gene expression of 14 proinflammatory cytokines such as IL-6, IL-1β, OPN, TNF-α, and RANTES was elevated more than 2-fold in the mock-infected aged mice as compared to the mock-infected young. Additionally, several interferon-inducible genes (CXCL9, CXCL10, and MX1) were upregulated in aged mice compared to young mice, in the absence of RSV-infection (Fig. S1A). Genes found upregulated in aged mice were then used to construct a GNCPro Pathway network (SABiosciences) that illustrates the relationships among the predominantly pro-inflammatory cytokines with TLR- and NLR-associated receptors (Fig. S1B).

To determine which antiviral genes are elevated or diminished in expression as a result of age in the setting of RSV infection, we isolated total lung RNA from aged and young mice infected with RSV A2 strain. This strain was selected based on extensive characterization of RSV A2 *in vitro*, which would provide a starting model for examining age-differential antiviral gene expression *in vivo*. The effect of RSV A2 infection on antiviral gene expression in young and aged mice was examined on days 1, 3, and 5 after infection. To minimize a noise in the data, we set threshold for gene selection of 2 fold and above and differential antiviral gene expression was observed between young (27 genes were up-regulated and 6 genes were down-regulated) versus aged mice (18 genes were up-regulated and 10 genes were down-regulated) (Table S1). This modulated gene list was used to analyze statistically significant Pathways. Among the top ten maps based on lowest p-values, identified from over 650 annotated signaling and metabolic maps, were ‘Innate immune response to RNA viral infection’, as well as ‘HMGB1/TLR signaling pathway’ (data not shown). The top ten relevant pathways shared among young and aged RSV infected mice involved predominantly cytokine signaling and TLR-activation. Interestingly, pathways towards IL-6, IL-1β, and MIP-1 signaling were upregulated in young; but induction of these pathways was diminished or absent in the aged. The map with the fourth lowest p-value was an immune response pathway ‘Histamine signaling in dendritic cells’ for the young category. In contrast, the fourth top-scored pathway unique among aged samples was ‘Inflammasome’ with a suggestive downregulation of the c-Jun/AP-1 transcription factors involved in inflammasome activation. Genes found >2-fold upregulated were used to construct a network map using the SABiosciences Gene Network Generator Pro and illustrates likely protein-protein or coexpression connections between the genes identified (Fig S1a &b).

**Figure 3:**
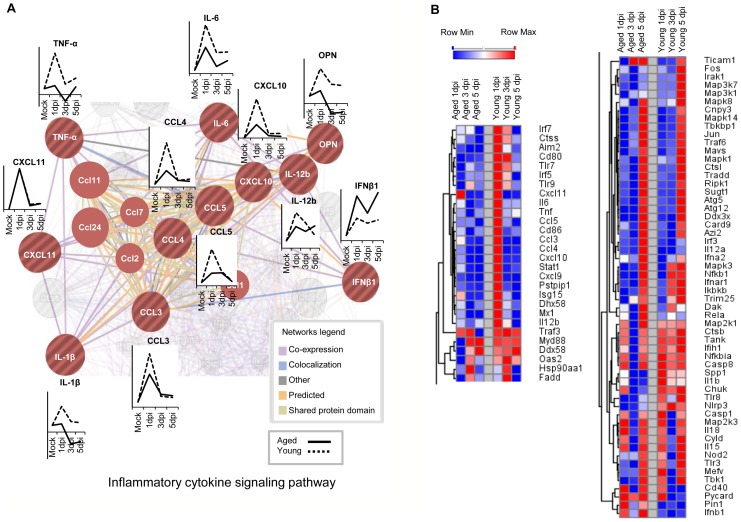
Pathway network and heatmap assembly identified age-related changes to the activation of proinflammatory cytokine signaling. Expression of 84 antiviral gene targets in A2-infected young and aged mice was compared with RT^2^-PCR Profiler array analysis. (A) 27 genes from the PCR array analysis were found upregulated >2-fold on 1 dpi; a GeneMania network map was generated using the 27 gene symbols and the most relevant biological pathway recalled involved cytokine signaling (11 genes are involved in such pathway from the 27 genes initially entered as input). The pathway illustrates the networks linking genes through co-expression (purple), colocalization (light blue), predicted interaction (orange), shared protein domains (light yellow), or other known relationship (gray). Above or below each of the 11 gene symbols is a relative gene expression chart displaying relative gene expression from either aged (solid) or young (dotted) over the time course of RSV A2 infection (1 dpi, 3 dpi, or 5 dpi). Fold-regulation changes derived by ΔΔCt calculation were in reference to age-matched mock-infected control group and illustrate age-related changes in gene induction kinetics. (B) Heatmaps encompassing the complete 84-gene PCR array were generated using GENE-E software where rows were sorted in ascending gene expression order and processed for hierarchical clustering using one minus Pearson's correlation. Gradients from blue to red indicate minimum to maximum expression of the indicated gene, respectively, within each row. Columns indicate the aged and young groups at 1 dpi, 3 dpi, or 5 dpi. The heatmap was separated into two for size: the first column illustrates genes upregulated in young mice 1 dpi and the second column clustered genes that are commonly upregulated in young and aged groups. The lower half of the second column illustrates a cluster of genes that remain upregulated on 5 dpi in young mice. Individual mouse RNA array analysis was performed in a single experiment with three different time points (n = 3 mice/group).

A GeneMania network map was then constructed to summarize the findings from the GeneGo pathway analysis (Fig. 3A). Despite fewer annotated pathways within the GeneMania database, the GeneMania pathway maps shared similar identity with the GeneGo analysis (data not shown). The 27 genes found to be upregulated >2-fold upon RSV infection in young mice at 1 dpi were used as inputs for GeneMania interaction pathway interaction analysis, where it was predicted that 11 of the 27 genes are associated with inflammatory cytokine signaling (Fig. 3A) with a low false discovery rate (FDR) of 8.69e^−24^ (coverage spanned 16 of 88 genes associated with cytokine signaling) [34]. Relative age-specific induction of antiviral genes between aged (solid) and young (dotted) was compared to age-matched mock-infected controls and displayed as miniaturized line graphs alongside each of the 11 genes. In all but one of the targets, young mice had greater induction at 1 dpi or 3 dpi. When comparing fold-changes in age-matched normalized gene expression, magnitude and kinetics of antiviral gene induction were affected by substantially by age. Heatmaps were assembled using GENE-E Heatmap software using the fold-change values obtained from the PCR array analysis; genes were then sorted for hierarchical clustering using one-minus Pearson’s correlation (Fig. 3B). RSV infection in young mice induced more innate antiviral gene expression earlier in infection, between 1 and 3 dpi, as seen in the first cluster on the column on the left. Innate antiviral gene expression levels generally tapered down by 5 dpi in young mice, with exception of a minor cluster of NLR- and TLR-associated genes, shown on the bottom-half of the second column; these genes remained elevated at 5 dpi. Alternatively, RSV infections in aged mice are diminished for the majority of the TLR- associated genes, and induction often peaks much later during infection at 5 dpi. It should be noted, however, that the heatmap illustrates relative induction of an individual gene over time after infection in each age group, even if the change of expression not found to be statistically significant.

### Statistical analyses identify biologically relevant genes that are altered by age and infection

**Figure 4:**
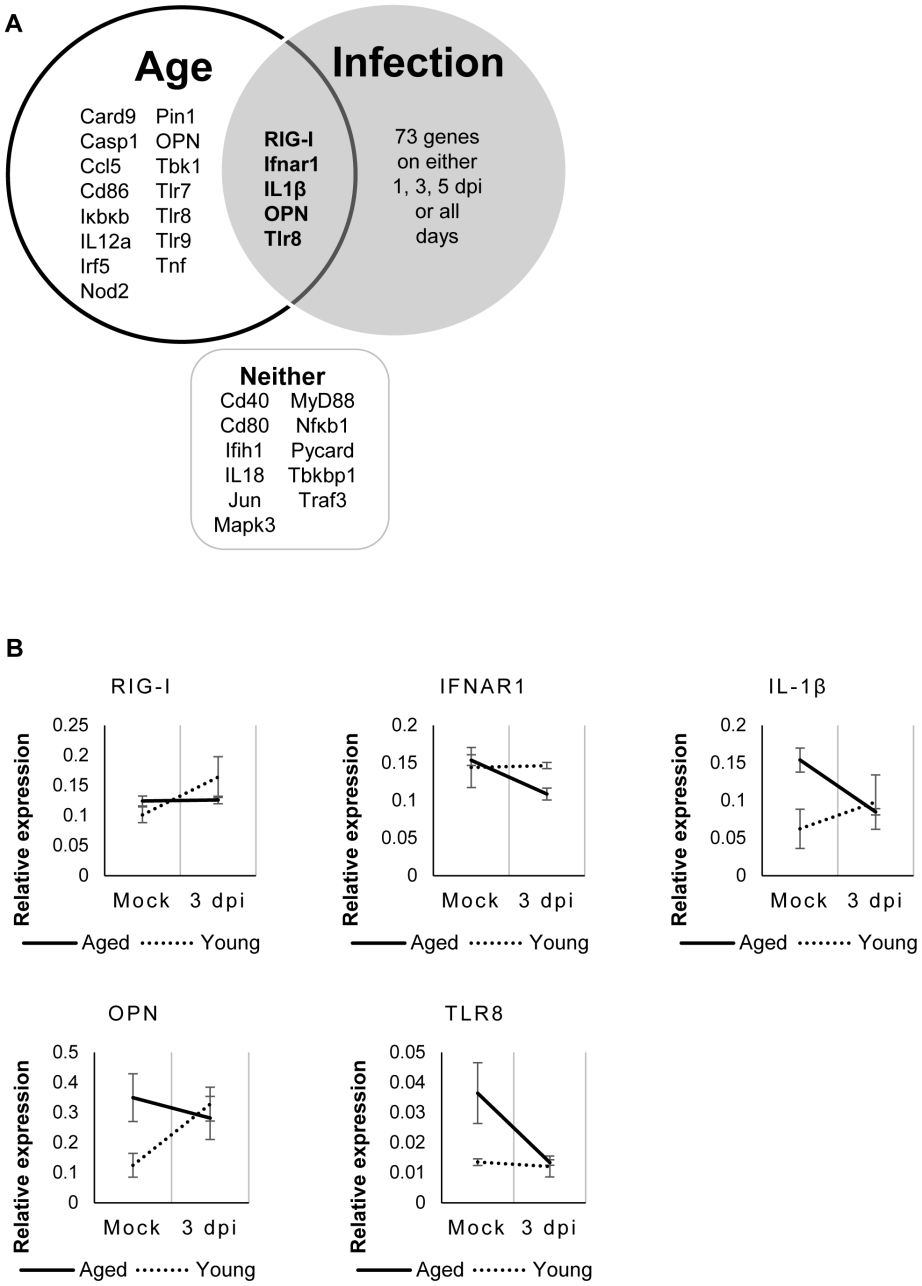
Five significant genes identified after DOE analysis. Minitab design of experimental analysis identified candidate genes that are significantly associated with age and/or infection on either 1 dpi, 3 dpi, 5 dpi, or combination of all days (p<0.05). (A) A Venn diagram illustrates these relationships and the significant genes associated only with age, infection, a combination of both factors, and neither on 1 dpi, 3 dpi, or 5 dpi. (B) Genes found significantly influenced by age and infection were used to construct individual graphs at 3 dpi and illustrate differences in direction and magnitude of gene expression of the five significant genes (*RIG-I*, *IFNAR1*, *IL-1β*, *OPN*, and *TLR8*) associated with both age and RSV A2 infection between young (dotted) and aged (solid) mice.

To identify which genes are uniquely regulated by the contributing factors of age or infection, a Minitab design-of-experiments (DOE) was performed on the first delta Ct derived from the PCR array analysis. Age was found to be a statistically significant factor influencing the ΔCt in 15 genes and the Venn diagram illustrates the relationship of the contributing factors of age or infection for all 84 antiviral genes examined (Fig. 4A). Several of the identified genes are associated with Nod-like receptor signaling and activation of inflammasome complexes, such as *Casp1*, *Casp9*, and *Nod2*. Age and infection were found to be statistically associated with five genes, *RIG-I*, *IFNAR1*, *IL-1β*, *OPN*, and *TLR8* and relative gene expressions from young and aged mice show age-related differences in the gene induction on day 3, when the change in ΔCt and subsequent relative gene expression was found to be statistically significant, often with contrasting trends between mock and 3 dpi (Fig. 4B). Of interest, 11 genes were not found to be significantly influenced by RSV infection over days 1, 3, or 5, including *CD40*, *CD80*, *IFIH1*, *IL-18*, *JUN*, *MAPK3*, *MyD88*, *NFKB1*, *PYCARD*, *TBKBP1*, and *TRAF3*. For the remainder of the study we focused on cytokines, OPN and IL-1β (upregulated >3-fold after infection in young mice) because of their associated roles in leukocyte migration and inflammasome activation, respectively.

### TLR7/8 signaling is impaired in aged alveolar macrophages

**Figure 5:**
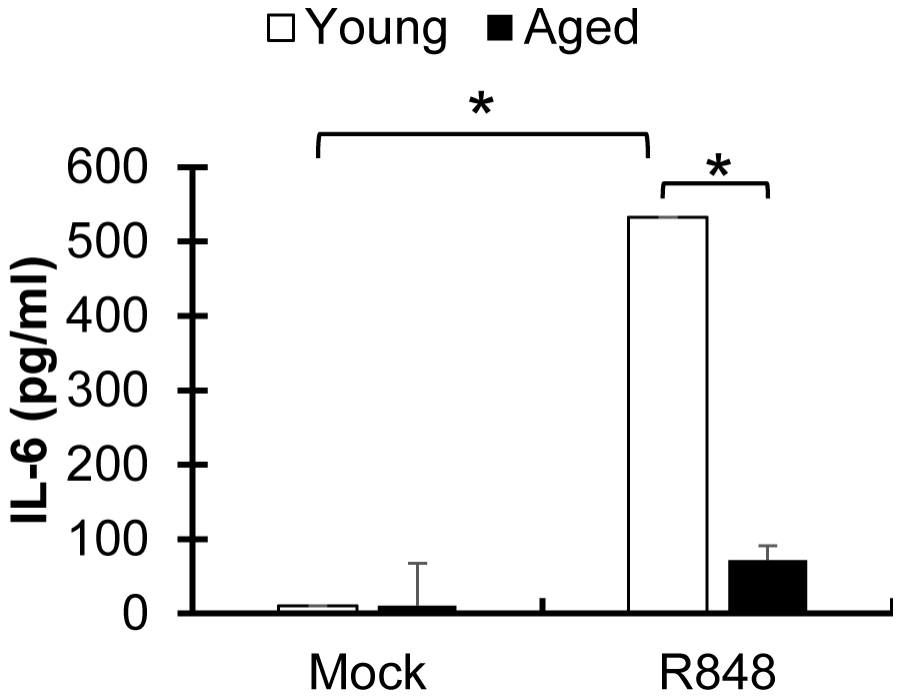
TLR7/8 activation is impaired in aged alveolar macrophages. TLR7/8 activation was examined in alveolar macrophages collected from uninfected young and aged BALB/c (n = 4 mice/group). Primary alveolar macrophages harvested from BALF were seeded at a density of 8×10^5^ cells per well in a 24-well plate and incubated with 2.5 µg/ml of the TLR7/8 ligand, R848, for 20 hrs. Culture supernatant was examined for IL-6 using ELISA to indicate TLR7/8 activation. Significance was determined with ANOVA 2-way analysis and experiment was performed in triplicate.

Since RSV-stimulated induction of TLR-associated genes appears diminished due to age and because of its established role in RSV-induced mucogenesis [16] and antiviral signaling, we wanted to test whether TLR7/8 signaling remains functional in aged mice. Alveolar macrophages were isolated from naive mice and stimulated *ex vivo* with the TLR7/8 agonist R848 for 20 h (Fig. 5). In response to R848, macrophages from young mice produced high levels of IL-6. In contrast, a significant reduction in IL-6 secretion was observed in aged alveolar macrophages after stimulation; IL-6 was undetectable in nonstimulated, age-matched controls. Secretion of IL-1β upon R848 stimulation was also examined because it may be secondary indicator of TLR-induced macrophage activity [35] but was found below detection in this study.

### RSV Infection in aged mice leads to altered kinetics of IL-1β

**Figure 6:**
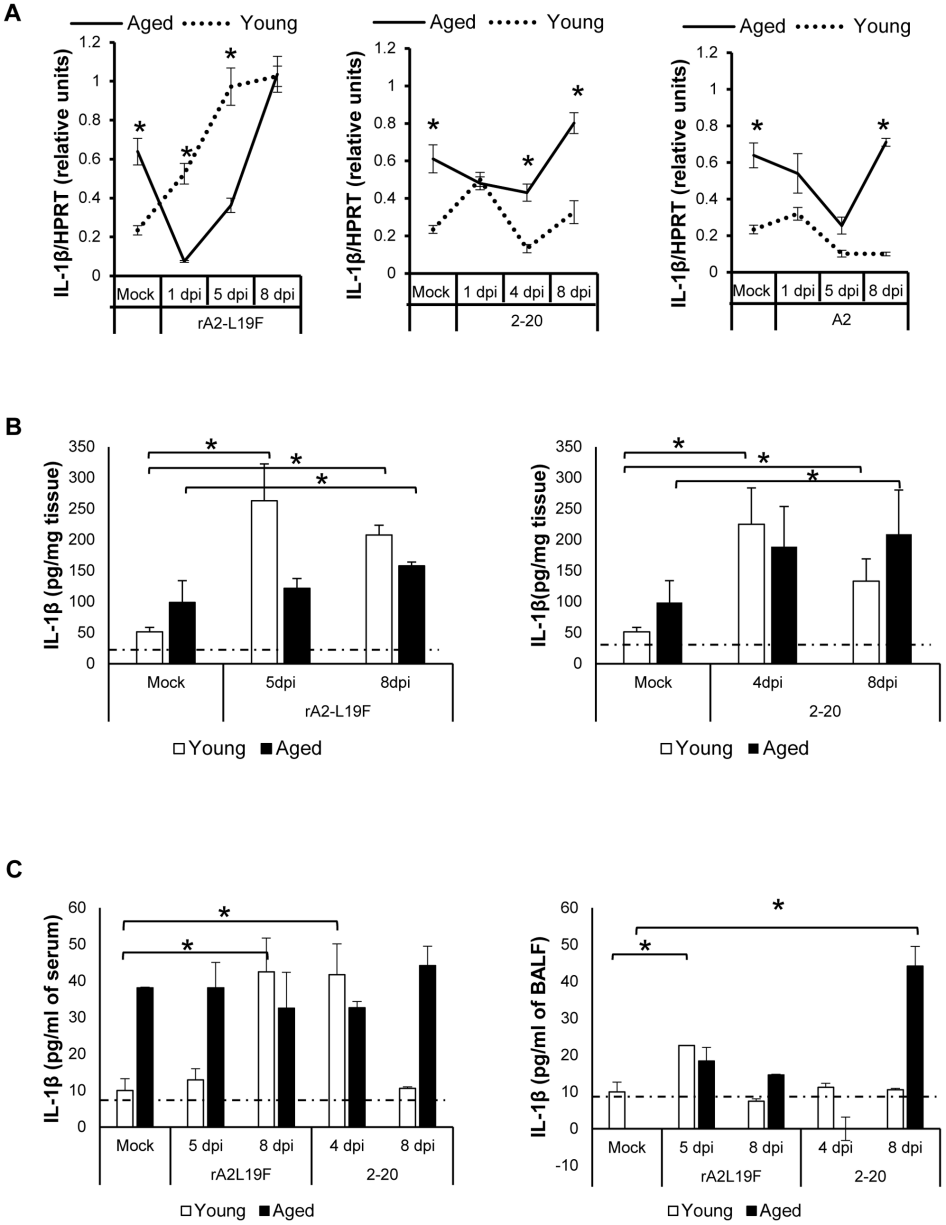
Age-differential gene expression of IL-1ββ in tissue, plasma, BALF after RSV infection. Young and aged BALB/c mice (n = 4/group) were intranasally infected with a dose of 10^6^ pfu/mouse of A2 or 10^5^ pfu/mouse of rA2-L19F or 2-20 and total lung RNA was collected on time points indicated. (A) RNA transcripts of IL-1β were analyzed with qRT-PCR and represented as a relative ratio of target gene expression to endogenous mouse HPRT and examined with rA2-L19F, 2-20, or A2 at various timepoints. (B) Lung tissue collected and homogenized for plaque assay was used for protein analysis using standard ELISA. Levels of IL-1β from rA2-L19F and 2-20 infected young and aged mice were obtained in pg/mg of starting wet-lung tissue. Levels of IL-1β were unchanged in A2 RSV infections and are not shown. (C) Levels of IL-1β in serum and BALF were analyzed using standard ELISA. Serum and BALF were obtained from individual mice and not pooled (n>4 per group). Horizontal line indicates the limit of detection using the ELISA method. Data are represented as means ±SEM. Experiments were performed in triplicate.

We were interested in comparing antiviral gene induction observed with moderate dose of A2 with more virulent RSV strains that induce more severe pathology and mucin production in BALB/c mice [26]; therefore qRT-PCR was performed on individual aliquots of total lung RNA from mice infected with either moderate dose of non-mucogenic A2 (10^6^ pfu/mouse) or low dose (10^5^ pfu/mouse) of mucogenic RSV strains rA2-L19F or 2–20 (Fig. 6A). Gene expression of mouse IL-1β was normalized to endogenous mouse HPRT mRNA levels. IL-1β mRNA levels are significantly elevated in aged mice as compared to young in the absence of infections. Upon RSV rA2-L19F-infections, IL-1β is diminished in aged mice compared to young on days 1 and 5 dpi; by 8 dpi, levels are comparable between age groups. Conversely, IL-1β mRNA levels were similar between young and aged on 1 dpi with RSV strains 2–20 and A2, but by 8 dpi with either strain, gene expression of IL-1β in aged mice of exceeds that of young. Of interest, IL-1β mRNA expression from aged mice infected with RSV 2–20 and A2 dropped at 4 dpi or 5 dpi; these occurred inversely proportional to maximum peaks of RSV N transcripts.

Since the precursor IL-1β isoform requires processing prior to being bioactive and functional, lung tissue was examined for levels of IL-1β using ELISA. RSV strains rA2-L19F and 2-20 infections were examined because they induced severe lung pathology in mice (Fig. 6B). Young mice produced significant levels of IL-1β upon infection with either rA2-L19F and 2-20 by 4 or 5 dpi; levels remained significantly elevated at 8 dpi. In contrast, levels of IL-1β in aged mice at 5 dpi remained unchanged. By 8 dpi, IL-1β was significantly elevated among both age groups and with either RSV infection. To assess local inflammation and cytokine release during RSV 2-20 infection, we performed immunohistochemistry on lung sections at 4 dpi for detection of IL-1β (data not shown). Number of IL-1β-positive cells was slightly increased in the airways of young mice after RSV infection at 4 dpi; but a change was less apparent in aged mice. Serum and BALF from mock- or RSV-infected mice were then compared to determine whether RSV infections lead to changes in either local or systemic inflammatory responses (Fig. 6C). In contrast to young, aged mock-infected mice have a higher baseline expression of IL-1β in serum, although levels were often below detection in BALF similar amounts total protein, quantified with BCA assay. IL-1β increased in both the serum and BALF of young mice after RSV rA2-L19F; however, the temporal increase in IL-1β were different between rA2-L19F and 2-20 in young mice; levels of IL-1β remained unchanged in the serum of aged mice regardless of RSV strain. RSV infection with rA2-L19F led to significant increase in IL-1β in young mice; increase was observed in aged mice but was not significant. Despite insignificant increases in BALF IL-1β upon 2-20 in young mice, aged mice had significant levels of IL-1β on 8 dpi. Similar to what we observed with mRNA levels, IL-1β production may be temporal during the RSV infection timecourse, as we observed a decline at 4 dpi in aged mice.

### RSV-infected aged mice have chronic inflammation but diminished local production of OPN in response to RSV infection

**Figure 7:**
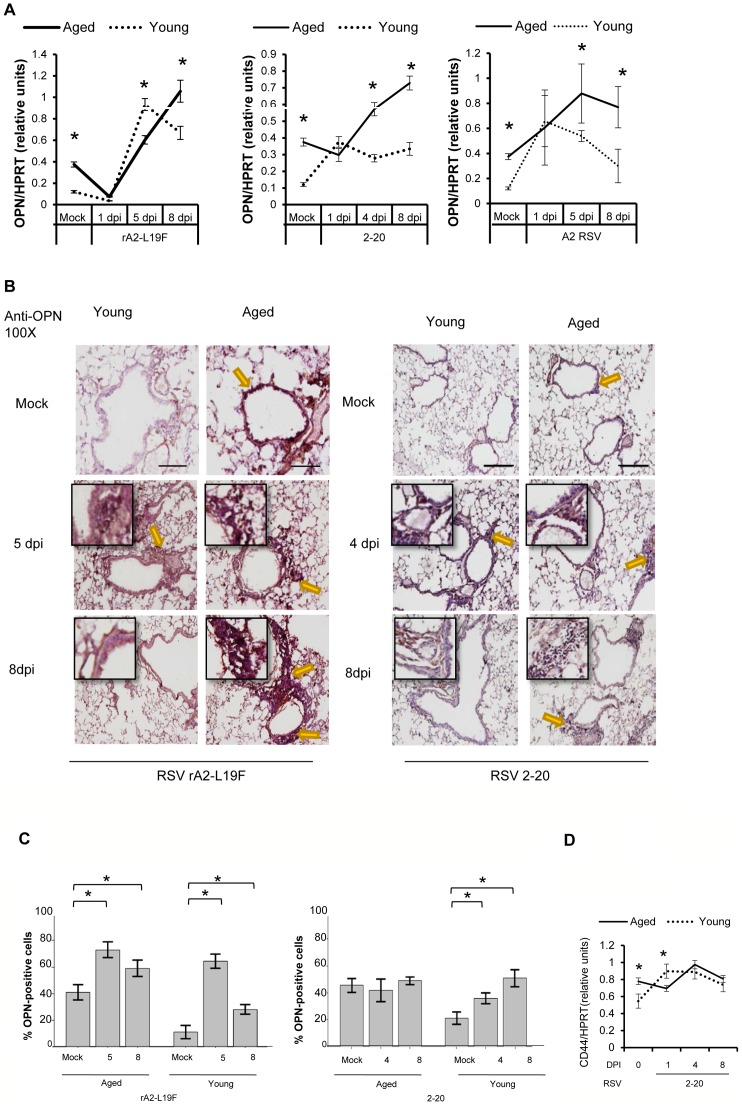
Aging results in diminished OPN production in response to 2-20 RSV infection. Young and aged BALB/c mice (n = 4/group) were intranasally infected with a dose of 10^6^ pfu/mouse of A2 or 10^5^ pfu/mouse of 2-20) and total lung RNA was collected on time points indicated. (A-B) RNA transcripts of OPN were analyzed with qRT-PCR and represented as a relative ratio of target gene expression to endogenous mouse HPRT and examined with rA2-L19F, 2-20, or A2. (B) 5 µm lung sections obtained at 4, 5 and 8 dpi with either rA2-L19F, 2-20, or A2 infected young and aged mice. Lung sections were immunostained for anti-mouse OPN and nickel-DAB reagent before counterstaining with hematoxylin and eosin. Representative images shown are at 100x magnification with inset [400x] displaying nickel-DAB (dark brown/black staining) positive cells and contrast the hematoxylin (light blue) nuclear stain. Representative images are shown with scale bar indicating 100 µm. (C) Enumeration of OPN-positive cells was performed with ImmunoRatio ImageJ analysis on 200X magnified lung sections from 8 dpi and values are shown as a percentage of total hematoxylin-stained cells in an individual box plot with mean interval bars. Within a single frame, at least 5 frames per mouse (n = 4/group) were collected and individual dot plots are shown of either aged or young mice with interval bars and significance, determined with ANOVA and Fisher's test (p<0.05). (D) qRT-PCR was performed on total lung RNA from young and aged 2–20 RSV infected mice for mRNA expression of OPN receptor CD44. Statistical significance was determine with ANOVA 2-way analysis with p<0.05. All experiments were performed in triplicate.

Gene expression of OPN was similarly examined with qRT-PCR and normalized relative to mouse HPRT. OPN expression was elevated in aged mice even in the absence of RSV, and by 8 dpi with any of the three RSV strains, the OPN mRNA transcript levels in aged mice significantly exceeded that of young mice (Fig.7A). Despite elevated OPN at baseline and at 8 dpi, the slope of OPN mRNA transcripts between 1 dpi and 5 dpi is diminished compared to young. We also found OPN to be slightly elevated in the BALF of mock-infected aged mice compared to mock-infected young mice and upon 2–20 infection, although BALF levels of OPN were often low or undetectable with ELISA (data not shown). OPN increased in young BALF but did not significantly increased in aged infected mice; no statistical difference was found between levels of OPN in the serum of young and aged mice. Immunohistochemical analysis also demonstrated constitutively higher expression of OPN in the airways of elderly mice prior to RSV infection (Fig. 7B). Infection with rA2-L19F results in increased expression of OPN in both young and aged mice on 5 dpi; however, as compared to a 7-fold increase in OPN-positive cells, aged mice had a 50% increase (Fig. 7C). By 8 dpi with rA2-L19F, OPN levels decrease in young but not aged mice, although number of OPN-positive cells remain higher than baseline expression. In contrast, 2-20 infection in both young yielded fewer OPN-positive cells than with rA2-L19F infections; moreover, the number of OPN-positive cells enumerated from the lungs of 2-20 infected aged mice remained unchanged from baseline. This suggests an age-dependent differential responses to the RSV stains examined here. Since neither severe cell infiltration nor mucus production [26] is associated with RSV A2, OPN-levels in the lung were not examined in this study.

Since OPN-levels were unchanged upon 2-20 infection, we examined CD44, a known receptor for OPN [17]; in a pattern similar to that of OPN, CD44 was increased upon 2-20 RSV infection and gradually returned towards baseline expression in young mice (Fig 7D). In contrast, CD44 was significantly overexpressed in aged mice compared to young, mock-infected mice, and showed dysregulated expression upon 2-20 infection, peaking at 4 dpi instead of 1 dpi; although the difference from young mice was not statistically significant. This comparative study of aged and young mice may provide insight as to why OPN-related signaling is impaired in aged mice upon RSV infections.

### OPN-/- mice have enhanced mucogenic responses to rA2-L19F infection

**Figure 8:**
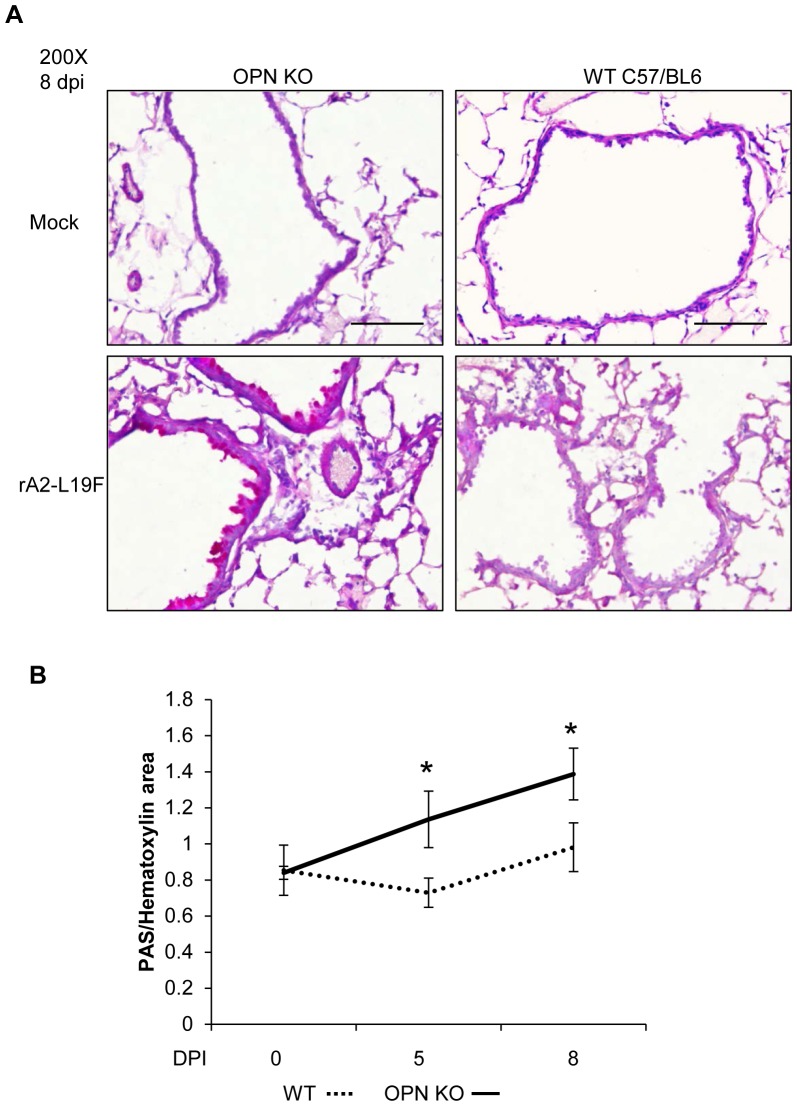
Mucus secretion in response to rA2-L19F is altered in OPN-/- mice. WT C57BL/6 and OPN-/- mice were intranasally infection with a high dose of mucogenic rA2-L19F (1×10^6^ pfu/mouse, n = 4) or a mock-inoculum, and at 5 and 8 dpi tissues were harvested. (A) Lung sections (5 µm) were stained with PAS per the manufacturer's instructions and counterstained with hematoxlin and eosin, then analyzed to calculate area of staining with ImageJ using Color Deconvolution plugin using single-stained customized vector settings. (B) The areas obtained through ImageJ analysis were related as a ratio of PAS-staining to hematoxylin staining, and displayed on a graph. A minimum of 10 fields of view were obtained at 200X per mouse and the images shown are a representation of the images collected. The experiment was performed in triplicate and data is displayed as means ±SEM.

Our observations that chronic OPN in aged mice was associated with enhanced and prolonged RSV disease led us to suspect excessive OPN enhances RSV disease in the mouse. To examine this, we infected OPN-deficient mice (OPN-/-) with a high infectious dose of mucogenic RSV rA2-L19F and compared PAS-staining and histopathology with strain control mice, C57BL/6 (Fig. 8A). In the absence of infection, airways were similar and indistinguishable. Upon rA2-L19F infection, however, PAS-staining increased OPN-/- mice as compared to WT, which had moderate mucus secretion. Cellular infiltration, determined by enumeration of hematoxylin-stained cells along airways, was not statistically different between the two mice groups after infection, although infiltration densities were both were comparatively less than BALB/c after rA2-L19F infection (data not shown). PAS-staining was analyzed using color deconvolution analysis and area of PAS-stained regions were compared to hematoxylin area; ratios were plotted in a graph and shown in Fig. 8B. OPN-/- mice had increased PAS-staining as compared to WT mice, although no significant change was observed in weight loss or clinical observations (data not shown).

### OPN-/- mice have greater resistance to rA2-L19F infection

**Figure 9:**
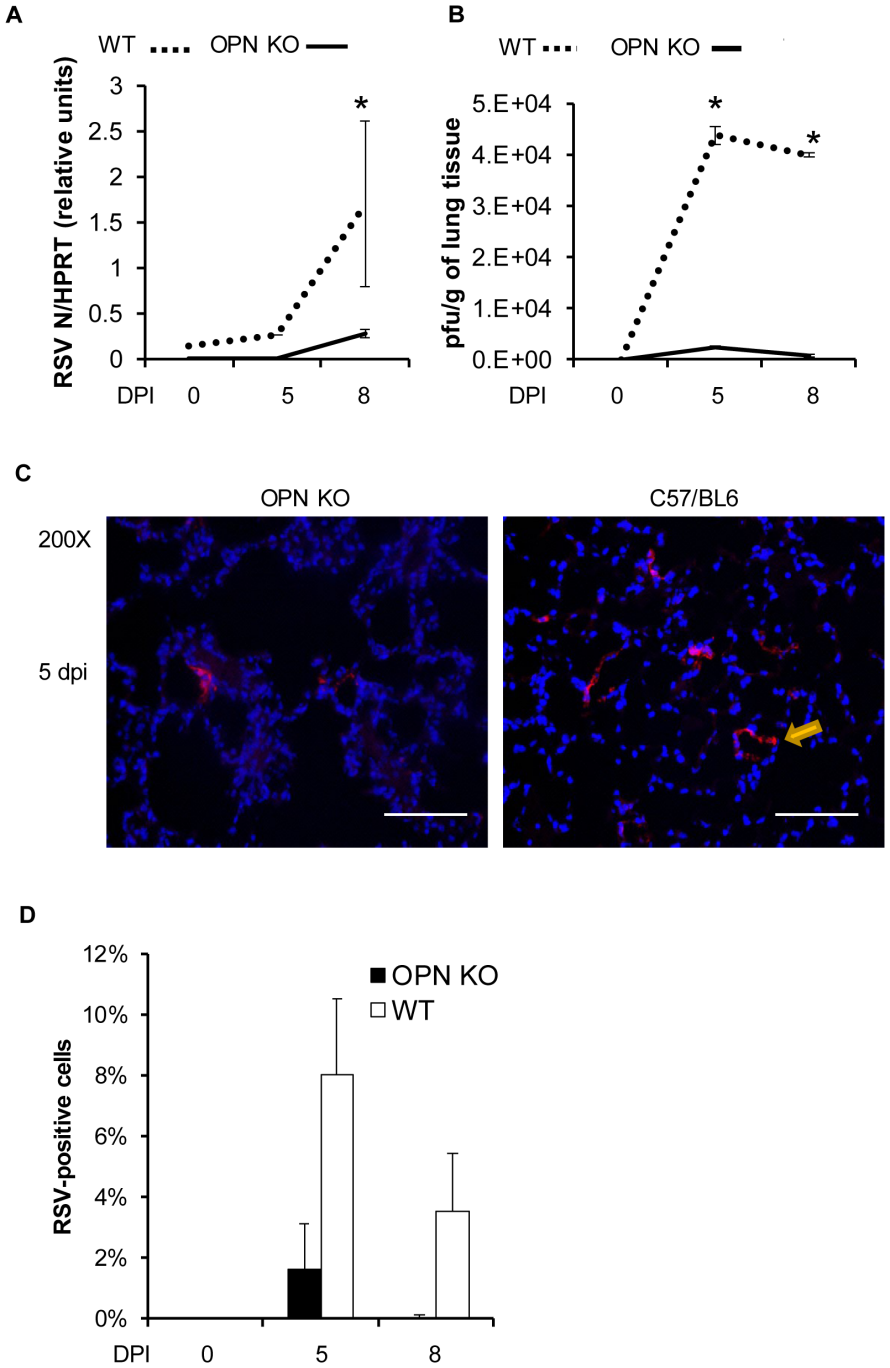
OPN-/- mice are protected from RSV infections. WT C57BL/6 and OPN-/- mice were intranasally infection with a high dose of mucogenic rA2-L19F (1×10^6^ pfu/mouse, n = 4) or a mock-inoculum, and at 5 and 8 dpi tissues were harvested. Total experiment was performed in triplicate, with data represented as means ±SEM. (A) Total lung RNA was analyzed with qRT-PCR for gene expression of RSV N and displayed as arbitrary relative units. (B) Plaques were obtained from the lung homogenates of infected mice at 5 and 8 dpi. (C) Lung sections (5 µm) were stained for the presence of RSV antigens using a polyclonal antibody and Alexa Fluor 555-secondary antibody and appears as red, while DAPI-stained nuclei appear blue. Images shown are representative of a minimum of 10 images collected at 200X magnification, all collected with identical exposure settings. (D) The number of RSV-positive cells was then calculated by ITCN ImageJ analysis, assuming differential diameters for quantifying the nuclei and individual cells. The percentage of RSV-positive cells is displayed as means ±SEM.

The evident increase in mucus secretion along the airways in OPN-/- after rA2-L19F infection was not originally anticipated, but we sought to determine whether deficiency in OPN also influences viral replication or viral disease. Infection of OPN-/- and WT with rA2-L19F was substantially lower in the mice strain that come from a C57BL/6 background (Fig. 9A). In comparison to BALB/c, RSV N gene expression peaked at 8 dpi as opposed to 5 dpi and expression as variable. Interestingly, WT mice had greater RSV N expression than OPN-/-. In addition, viral plaques were higher in lung homogenates from WT than OPN-/- on both 5 and 8 dpi (Fig. 9B). This was confirmed further with immunohistochemical staining of RSV antigens in the lung tissues (Fig. 9C); there were comparatively fewer RSV-positive lung cells in the OPN-/- mice than WT at both 5 and 8 dpi (Fig. 9D). The combination of reduced viral gene expression, lower plaque counts, and fewer RSV-positive cells in the lung sections indicate the OPN-/- mice had reduced infection as compared to their WT counterparts.

## Discussion

Despite the clinical significance and importance of aging in respiratory viral infections, current knowledge about aging and the antiviral immune response is still lacking. PRR signaling mediates recruitment of immune cells, control of mucogenic responses, and activation of antiviral factors, and yet the effect of aging on these pathways remain incompletely understood. A recent study found older age led to diminished antibody responses to RSV F antigens in comparison to young, although the capacity to generate neutralizing antibodies was not entirely absent in aged mice [24]. The addition of an adjuvant enhanced immunogenicity even in aged mice, suggesting the immune system in the aged are dampened, not abolished. In this study, we aimed to elucidate the differences in the ability to respond to virulent strains of RSV using an aged mouse model.

To identify early changes in innate responses, we chose to monitor antiviral gene expression between 1 and 5 dpi. RSV infection in aged BALB/c mice resulted in altered expression of PRR genes and inflammation *in vivo* as compared to young mice. GeneGo and GeneMania analyses identified statistically significant signaling pathways related to fold changes in gene regulation and correlated potential impaired inflammatory responses with aged mice. Minitab DOE identified key genes that are statistically associated with age or age and RSV infection. By examining the ΔCt as well as fold-changes in respect to age-matched controls, we observed noticeable age-specific differences in baseline innate antiviral gene expression as well as induction after RSV infection; cytokines and receptors associated with early innate immunity were examined in this study and not adaptive responses since a previous investigation already examined age-associated changes to adaptive immunity to RSV [22]. Five genes statistically associated with both RSV and age were RIG-I, IL1-β, IFNAR1, OPN, and TLR8, which likely play important roles in defending against early RSV infections; further investigation of these genes may help serve in the development of age-specific therapeutics.

Altered kinetics of these innate immune genes may delay activation of downstream antiviral mediators and contribute to the exacerbation of disease or poor adaptive immunological responses, characteristics which are often associated with older age [12], [14], [36]–[40]. Diminished expression of TLR8 on cultured monocytes from infants was previously correlated with impaired early antiviral responses to RSV and TNF-α production [41]. The role of IFNAR1 and RIG-I in IFN induction has been well studied, but the mechanisms of RIG-I-mediated production of IL-1β in RSV infections remain unclear. There is the potential for the diminished RIG-I signaling associated with age to subsequently prevent IL-1β production after RSV infection [42], however this remains to be investigated. Diminished functionality of the early innate antiviral system potentially leads to enhanced inflammation and altered inflammatory responses and we observed aged alveolar macrophages had diminished ability to respond to TLR7/8 stimulation. We suspect that the impairment of local secretion of inflammatory cytokines by immune cells, such as alveolar macrophages, delays defenses against RSV and consequently prevent the ability to promptly recruit cells in early infection (1 dpi) and clear functional immune cells at later time points.

Histopathological analysis of lungs from mucogenic RSV-infected mice similarly demonstrated age-dependent immunopathology and delayed leukocyte clearance in aged mice. In contrast to previous reports and A2 infection, viral clearance in rA2-L19F and 2-20 infected mice was substantially delayed in aged mice, suggesting more severe pathology can be attributed to both age as well as virus pathogenicity. Since IL-1β and OPN were statistically significant during early time points in A2 infection (1–3 dpi) and associated with older age, we examined them further at 5 and 8 dpi with mucogenic RSV strains in lung tissue and fluids. Aged mice constitutively expressed more IL-1β and OPN than young mice in the absence of infection and similar hyperinflammatory responses are reported to occur in elderly humans[43]; upon exposure to a new pathogen trigger such as the RSV infection, IL-1β and OPN production was delayed and prolonged, suggesting distinct impairments in the activation of antiviral responses.

Importantly, IL-1β is recognized as a by-product of inflammasome activation and is necessary for rapid neutrophil recruitment and viral clearance [44]. Impaired IL-1β induction may delay early antiviral responses and contribute to cellular accumulation late in infection. It should be noted, however, that pro-IL-1β requires processing before the active form can serve function in signaling and RNA analysis does not necessarily correspond with bioactive IL-1β; we observed intriguing age-related differences in protein levels of IL-1β upon infection in lung tissue, BALF, and serum, although IL-1β was often in such low quantities it was below the detection threshold. Levels of IL-1β mRNA were generally higher in elderly mice but in tissue, serum, and BALF, protein levels were generally less than young. Potentially, aging could result in incomplete or impaired IL-1β precursor processing upon stimulation, thus explaining the decline in active IL-β despite elevated mRNA levels, however this is unlikely since an investigation suggests the NLRP3 inflammasome and its activation by ATP, not the processing of IL-1β precursor, is impaired in aged bone-marrow derived macrophages[45].This further supports our findings that the activation and regulation of inflammatory cytokines and downstream activities are impaired as a result of aging.

Also, our data showed an association of OPN with enhanced RSV disease, to our knowledge for the first time. The mechanism of OPN-mediated increases in RSV however remains unclear. We also found corresponding kinetics of the expression of receptor to OPN, CD44. A variety of immune cells secrete OPN, including activated T-cells, dendritic cells, and importantly, macrophages secrete OPN upon TLR stimulation, which may negatively regulate IFN-β production [46] and suppress IL-10 [20]. Conceivably, dysregulated or chronic OPN production in elderly mice could result in excessive leukocyte and monocyte/macrophage migration but negatively regulate antiviral responses. Our data indicate OPN-deficiency led to enhanced mucus secretion and reduced viral loads in the lung; this resembles the phenomena observed when IL-13 [47] and MUC5AC [48] are overexpressed in mice and the animals are protected from viral infection. Mucus secretion likely assists with prevention of viral spread, although excessive mucus leads to obstruction and pathology [16], [28]. Thus, chronic or dysregulated OPN production may be disadvantageous for elderly individuals and increase their susceptibility to RSV.

Also our data shows that the deficiency of OPN attenuates RSV infectivity in young mice; which produce relatively less mucus. This result suggests that there may be mucus-independent effects on RSV infections, the mechanisms of which remain to be elucidated. OPN was considered necessary, if not essential, for the defense against microbial biofilm production by oral pathogens[17], [49]. Deficiency in OPN increases susceptibility to herpes simplex virus and is required for early neutrophil recruitment in *Klebsiella pneumoniae* infections [17]; and OPN also regulates influenza infections, though it remains unclear whether unregulated OPN exacerbates disease.

While our results show that there is increased OPN levels in aged mice, it is unclear whether OPN causes or accelerates aging or it is an effect of aging. Since OPN protects from cartilage degradation observed in osteoarthritis [50], the OPN does not appear to directly contribute to the aging process. Recently, inflammasome-mediated production of IL-1β was also reported to induce chemotactic factors in autoimmune encephalomyelitis, leading to upregulation of OPN [51]. New evidence strongly suggests aging contributes to the decline of inflammasome-directed antiviral responses [52] and chronic overproduction of OPN [53]. Agents that cause pulmonary tissue damage, including cigarette smoke, induce OPN expression, leading to an accumulation of pathogenic macrophages and pulmonary Langerhans cells [54] and has been associated with exacerbated lung diseases such as in cancer, lung fibrosis, and allergic asthma [55]. Transgenic mice overexpressing OPN exhibit altered cell migration and adhesion signals[46], [56]–[58]; defects in the cardiopulmonary, tissue repair, endothelial and immune systems, the latter including Th1 [18], CD8+ T and NK cells [59], [60]; and have higher death rates and increased incidence of malignant tumors. On the other hand, OPN-deficient mice demonstrate abnormal migration of leukocytes which results in altered wound healing and leukocyte migration, particularly in mouse models of bleomycin-induced pulmonary fibrosis [61] and airway remodeling[62]. In healthy adults, OPN production is tightly regulated and exclusively produced in response to cell injury; therefore, chronic overproduction of OPN in aged mice may indicate significant defects in immune responsiveness and signaling. This is supported by reports of aberrant inflammatory cytokine production as a consequence of the natural aging process, including IL-6, TNF-α [12], [63], [64], and in this study and others, IL-1β. Further examination is needed to better understand how increased inflammatory cytokines like OPN contribute to RSV-induced lung disease, particularly since NK cells contribute to acute lung injury [65], [66]. Despite reported age-related chronic production of OPN and role in leukocyte recruitment, bone remodeling, and immune cell activation, the effect of chronic OPN production on other diseases remains largely unstudied. Thus, there is paucity of evidence suggesting that OPN causes or contributes to the aging phenotype. Instead, we suspect chronic, dysregulated OPN production is likely a consequence of cumulative defects that arise during the natural aging process, and it alters immune and other cellular processes in response to new infections or exhaust ATP and signaling components involved in antiviral immunity.

Thus, in this study, we observed that aging alters expression of innate immune PRRs, IL-1β and OPN production, and leads to more severe RSV lung pathology. Consequently, we anticipate that potential antiviral therapeutics relying on fully functional innate responses will be less effective in elderly populations since there is evidence of significant deficits in the innate immunity. Treatments for RSV-induced lung disease will likely need to be tailored to treat chronic systemic inflammation and compensate for impaired local antiviral responses in the airway of elderly. Also, decreased RSV infection of OPN KO mice compared to WT suggests the potential of inhibiting OPN to treat RSV infection.

## Supporting Information

Figure S1 **Baseline expression of antiviral genes in absence of RSV.** Mock-infected aged and young mice were compared with the PCR array and analyzed with the Web-based RT^2^PCR Profiler Data PCR array analysis software. (A) A dot plot with logarithmic scale was generated with the PCR array analysis software (SABiosciences) and genes with greater than 2-fold upregulation are listed in the table insert. Genes with asteriks were found to be statistically upregulated in mock-infected aged mice as in comparison to mock-infected young mice. (B) The genes identified to be upregulated >2-fold were used to generate a network map using SABiosciences Gene Network Generator Pro.
(TIFF)

Table S1 **Normalized antiviral gene expression from aged and young mice.** RT^2^PCR Profiler Data PCR array analysis was performed on RSV A2-infected young and aged mice (n = 3) and normalized gene expression was derived using ΔΔCt calculations and the arithmetic mean of 5 endogenous housekeeping genes. Shown in Table S1 are the mean gene expression values >2-fold when normalized to age-mock controls. Red values indicate upregulation while blue indicates downregulation of genes. The total number of genes per timepoint are indicated at the bottom of the table.
(TIFF)
