# Supplementary figures and images for: Respiratory Syncytial Virus (RSV) Infection in Elderly Mice Results in Altered Antiviral Gene Expression and Enhanced Pathology
Source: PLoS One. 2014 Feb 18;9(2):e88764. doi: 10.1371/journal.pone.0088764 (PMC3928298; doi:10.1371/journal.pone.0088764)

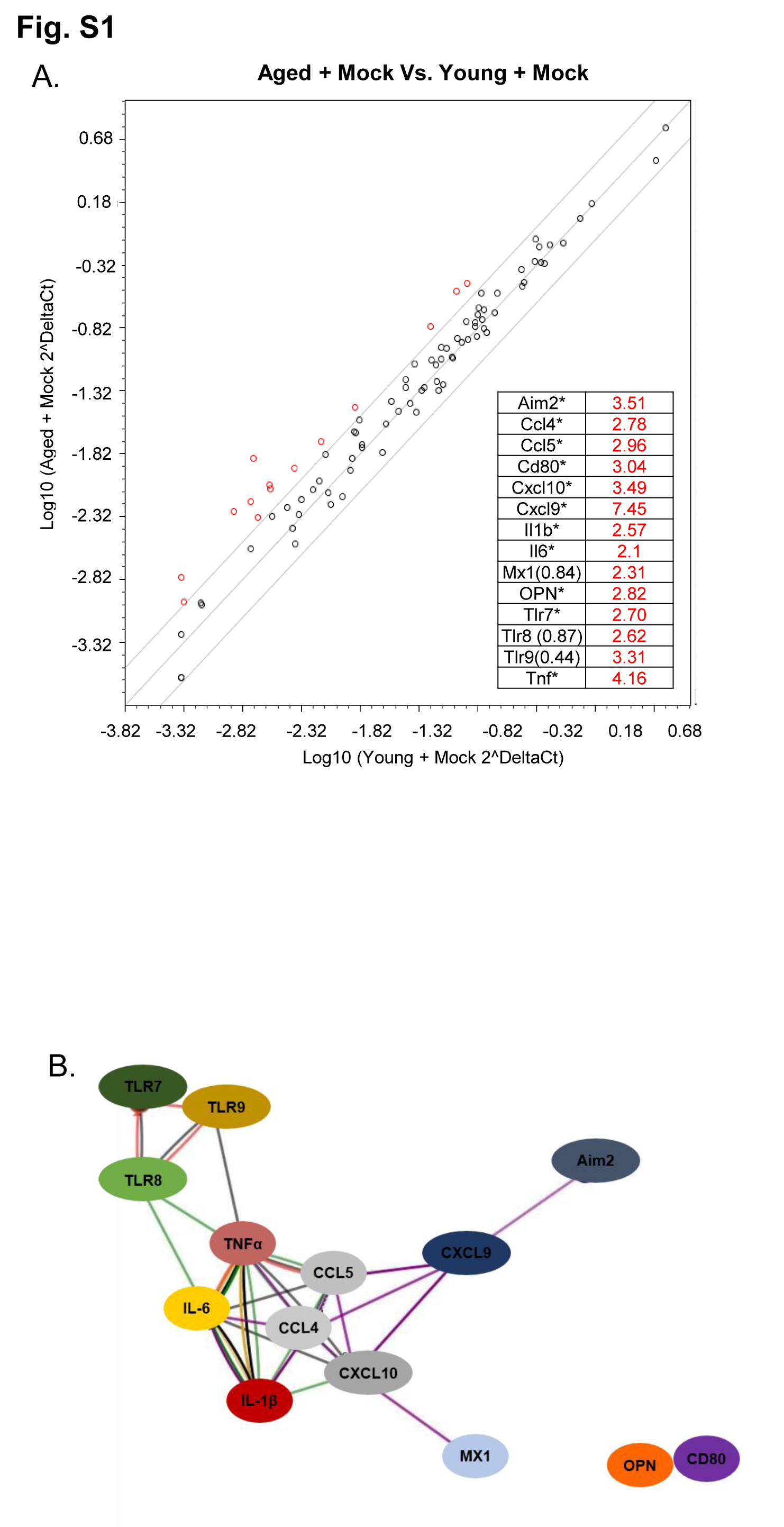

Supplement: Figure S1 — Baseline expression of antiviral genes in absence of RSV. Mock-infected aged and young mice were compared with the PCR array and analyzed with the Web-based RT2PCR Profiler Data PCR array analysis software. (A) A dot plot with logarithmic scale was generated with the PCR array analysis software (SABiosciences) and genes with greater than 2-fold upregulation are listed in the table insert. Genes with asteriks were found to be statistically upregulated in mock-infected aged mice as in comparison to mock-infected young mice. (B) The genes identified to be upregulated >2-fold were used to generate a network map using SABiosciences Gene Network Generator Pro. (TIFF) [file pone.0088764.s001.tiff]

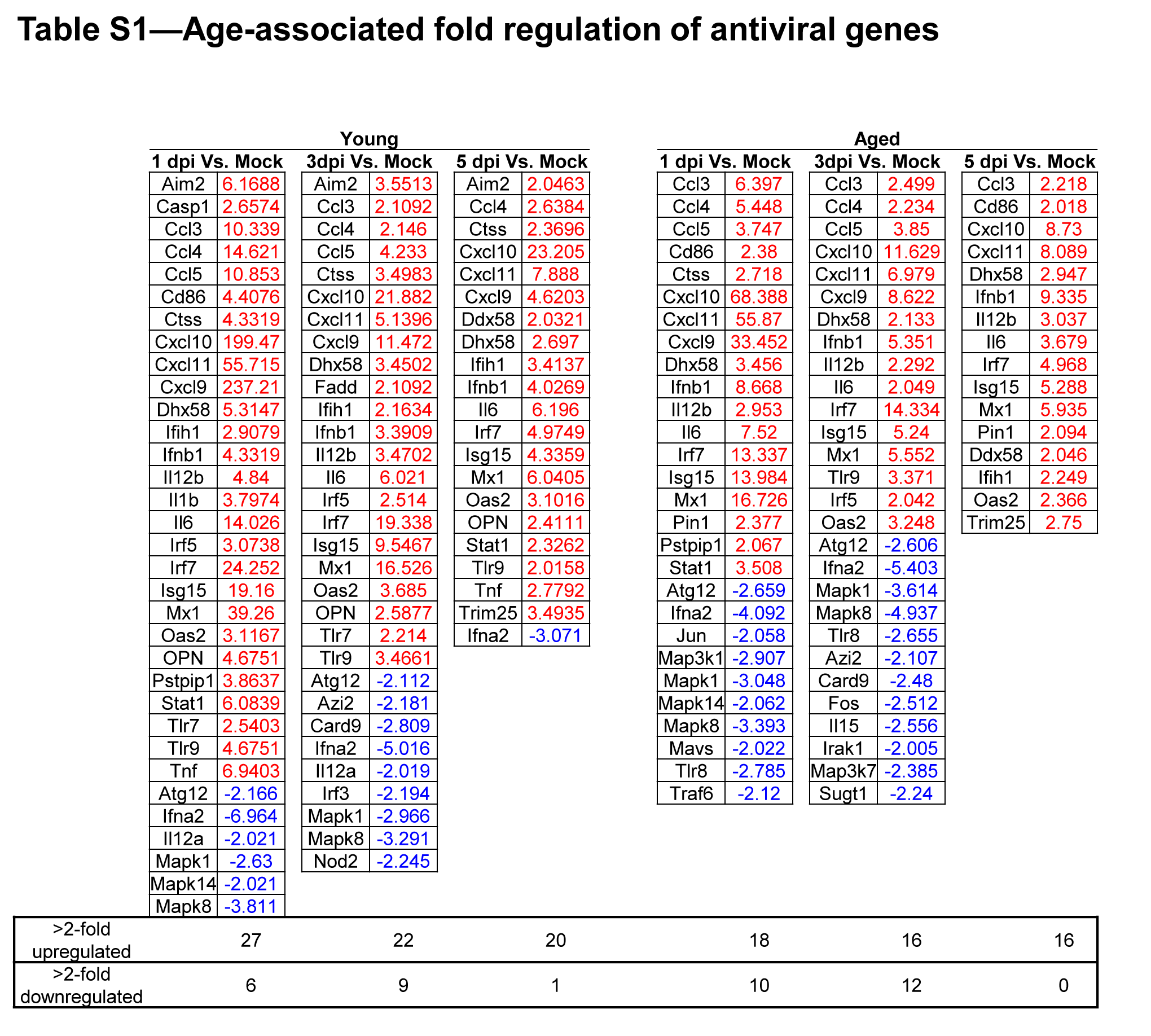

Supplement: Table S1 — Normalized antiviral gene expression from aged and young mice. RT2PCR Profiler Data PCR array analysis was performed on RSV A2-infected young and aged mice (n = 3) and normalized gene expression was derived using ΔΔCt calculations and the arithmetic mean of 5 endogenous housekeeping genes. Shown in Table S1 are the mean gene expression values >2-fold when normalized to age-mock controls. Red values indicate upregulation while blue indicates downregulation of genes. The total number of genes per timepoint are indicated at the bottom of the table. (TIFF) [file pone.0088764.s002.tiff]
